# Atrioventricular Synchrony Algorithm Modeling of a Leadless Pacemaker Family: A Virtual Patient Analysis

**DOI:** 10.1007/s13239-025-00783-0

**Published:** 2025-05-27

**Authors:** Miguel A. Leal, Todd Sheldon, Keelia Escalante, Mikayle Holm, Michelle Galarneau, Kurt Stromberg, Jonathan P. Piccini

**Affiliations:** 1 Emory University School of Medicine, Atlanta, GA USA; 2 Medtronic, Inc, Mounds View, MN USA; 3 Duke University Medical Center and Duke Clinical Research Institute, Durham, NC USA

**Keywords:** Algorithm, Atrioventricular synchrony, Leadless pacemaker, Modeling, Virtual patient

## Abstract

**Purpose:**

To assess the impact of enhancements to the Atrioventricular Synchrony (AVS) algorithms of a next generation Micra leadless pacemaker (Micra AV2).

**Methods:**

Accelerometer data were extracted from the AccelAV clinical study and were used to create virtual patients. A series of Monte Carlo simulations were run for each virtual patient to compare an enhanced Atrial Sensing Setup algorithm and Auto + A3 Threshold algorithm vs. original algorithms. A real-world survey was also conducted to observe clinical time savings from AVS programming burden reduction.

**Results:**

The enhanced Atrial Sensing Setup in Micra AV2 devices demonstrated > 70% AVS in 27 of 30 (90%) patients while 13 of 30 (43%) Micra AV patients had > 70% AVS (*p* < 0.001) with no manual programming. The Micra AV2 Auto + A3 Threshold without additional manual programming demonstrated improved overall ambulatory AVS in the 80–100 bpm range (84.1%). Based upon survey results, the enhanced Atrial Sensing Setup algorithm accounted for an estimated reduction in median device check time of 13 min per patient.

**Conclusions:**

Simulation-based analyses of the Micra AV2 leadless pacemaker projected significant improvements in automatic AVS at high sinus rates and an increase in the number of patients with > 70% AVS without clinician programming. Real-world survey results reported a reduction in device check time with the improvements. *Significance:* Improvements in the AVS algorithms in Micra AV2 allow for better automatic AVS at higher heart rates and reduced clinic utilization burden.

## Introduction

Leadless pacemakers were developed to overcome many of the complications associated with pockets and leads of traditional transvenous devices. While first-generation leadless pacemakers provided single-chamber ventricular rate responsive pacing only, the next generation, Micra AV, was developed to provide atrioventricular synchronous (AVS) pacing. Micra AV utilizes the accelerometer to detect atrial contractions and provides AVS for patients with AV block and intact sinus function [1–3].

**Fig. 1:**
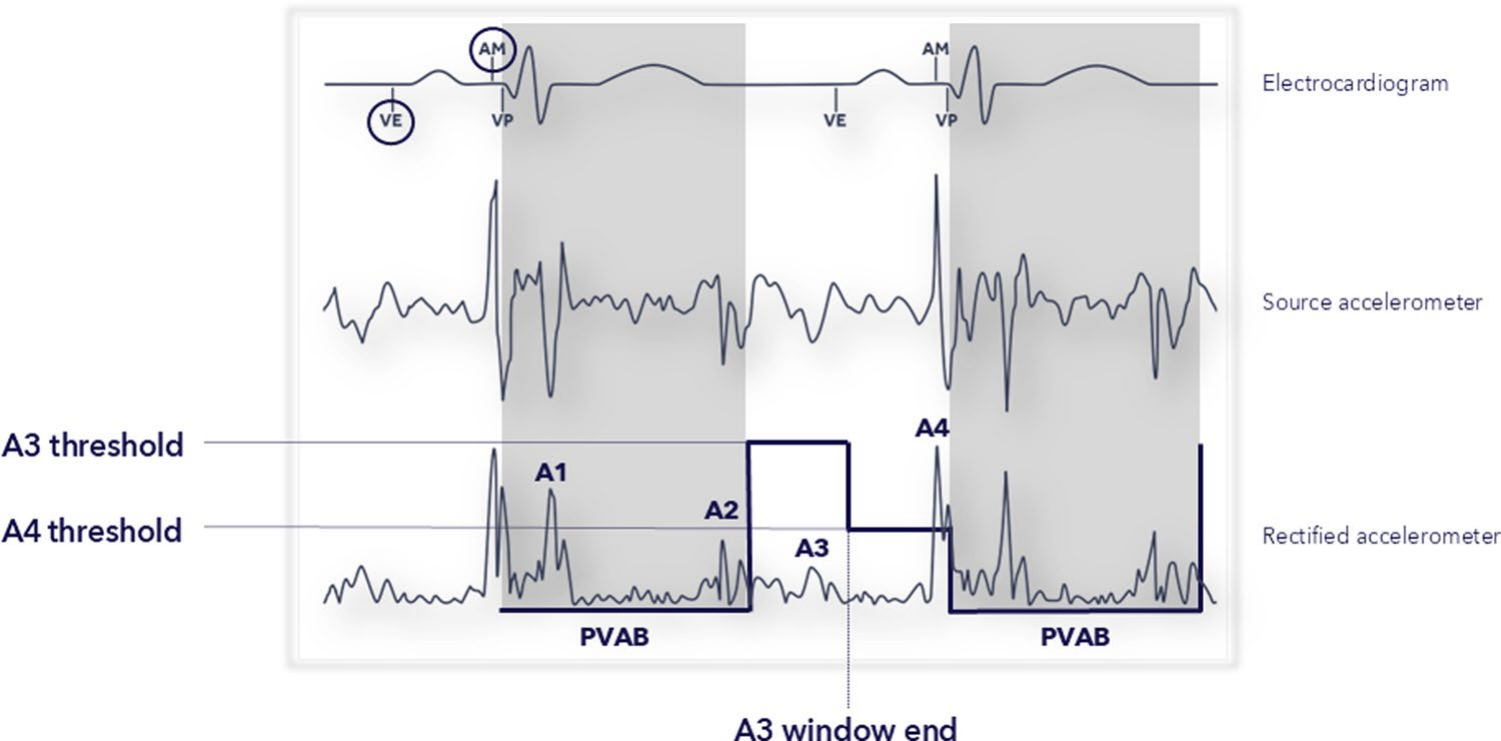
Atrial Mechanical Detection Algorithm. Visual depiction of how the Micra AV device uses the accelerometer signal to detect the atrial contraction (A4) and subsequently deliver AV synchronous pacing. The A3 Threshold is set above the max A3 signal, which is the signal occurring in the A3 detection window. The A4 Threshold is set below the max A4 signal, the signal occurring in the A4 detection window, as the aim is to detect the A4 signal (atrial contraction). The A3 Window End parameter separates the A3 and A4 Thresholds. The Ventricular End (VE) marker indicates the end of the A1–A3 ventricular event signals. The Atrial Mechanical (AM) marker indicates the device detected atrial mechanical contraction or A4. The Post-ventricular atrial blanking (PVAB) period is when the A1 and A2 signals are blanked. No atrial sensing occurs during PVAB. *VP* ventricular pace. ©Medtronic. 2024

The accelerometer signals measured in the ventricle by the Micra AV device are denoted A1, A2, A3, and A4, analogous to the heart sounds characterized as S1-S4 (Fig. 1). The key to achieving AVS with this device is to detect the atrial contraction (A4), while not detecting the passive ventricular relaxation signal (A3). The detection becomes more complex at higher sinus rates (typically starting at 80 bpm) when the A3 and A4 signals superimpose. The superimposed signal is denoted A7 (A3 + A4). A detailed description of the algorithm has been published previously [3]. Briefly, the A3 Threshold is set above the A3 signal but below the A7 signal. The A3 threshold algorithms automatically adjust the A3 Threshold as needed. The A4 Threshold is set below the A4 signal, as the aim of the Micra AV device is to detect the A4 signal (atrial contraction). The time at which the higher A3 Threshold switches to the lower A4 Threshold value is denoted as the A3 Window End parameter.

The AccelAV clinical study was designed to assess AVS in Micra AV patients over time. The study demonstrated that AVS was stable during both a 1-month and 3-month assessment and that a mean ambulatory AVS of 82.6% over a 24-h period could be achieved [2]. However, this AVS performance required clinician intervention to program the detection algorithm. The need for reprogramming of the detection algorithm is consistent with multiple studies that have assessed AVS [2, 4–6]. The A3 Window End parameter was often too long in patients following the Atrial Sensing Setup algorithm. Therefore, there was a need for an improved automated parameter setup algorithm aimed to reduce the need for reprogramming and decrease clinician burden.

**Fig. 2:**
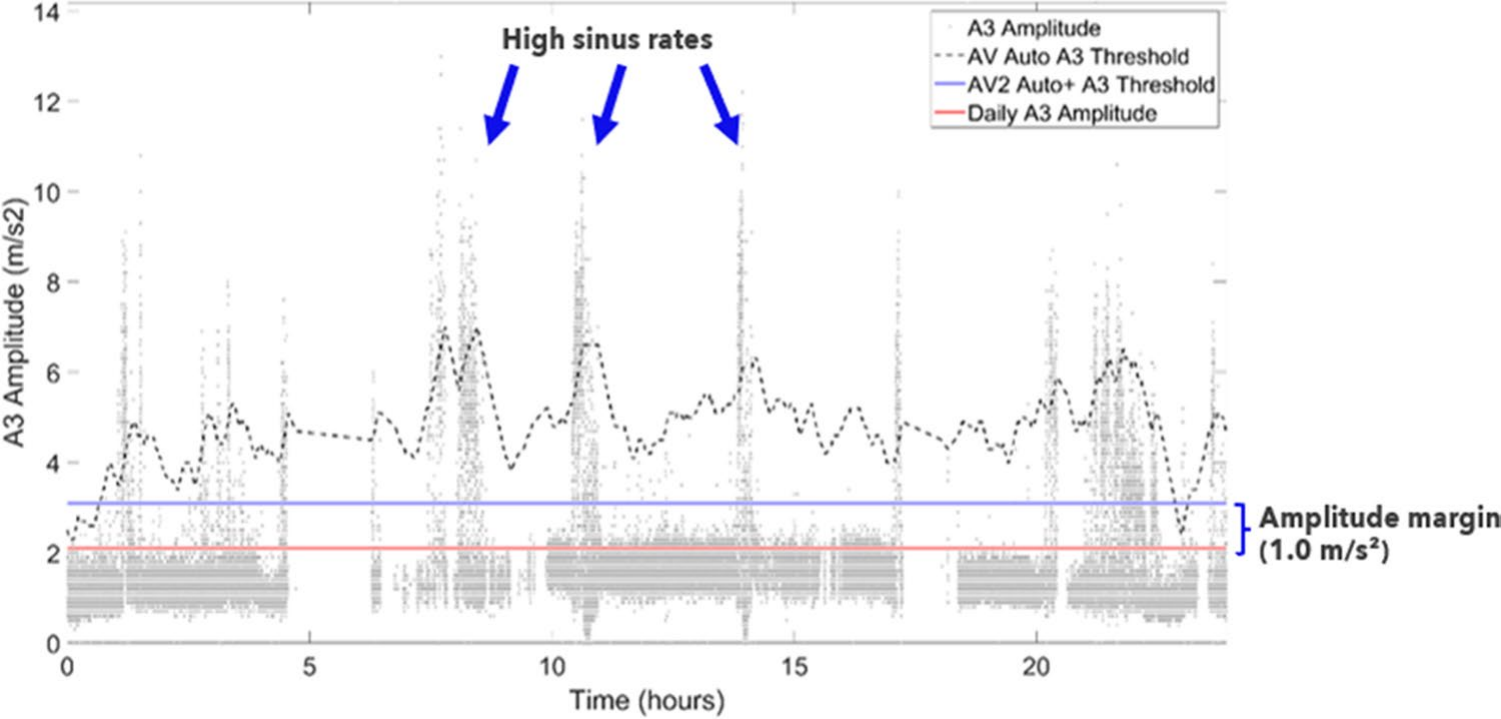
24-h recording from an AccelAV patient illustrating the A3 operation. The individual dots are the A3 amplitude measured by the device on each cardiac cycle. Periods of large A3 amplitude measurements are during high sinus rates when the A4 signal occurs in the A3 window, resulting in a large, combined A7 signal. The A3 amplitude measurements of 2.0 m/s^2^ and lower are during periods of lower sinus rates where the isolated A3 signal is measured. The dotted black line represents the Micra AV Auto A3 Threshold operation and during these periods of high sinus rates, the A3 Threshold adjusts too high resulting in undersensing. The red line is the Micra AV2 measurement of the A3 amplitude and is 2.1 m/s^2^. The Micra AV2 Auto + A3 Threshold then sets the new A3 Threshold at 3.1 m/s^2^ with the nominal A3 Amplitude Margin of 1.0 m/s^2^

The AccelAV clinical study included a sub-study, called Optimize, that sought to assess AVS with expert programming. The main programming change in the AccelAV Optimize sub-study involved turning Off the Auto A3 Threshold algorithm, because the Micra AV Auto A3 Threshold often adjusted too high during sustained high sinus rates (typically greater than 80 bpm) and programming a manual fixed A3 Threshold kept the A3 Threshold in an appropriate place to sense the A7 signal at higher rates (Fig. 2). An automated A3 Threshold algorithm that learns a patient’s isolated A3 signal and programs an appropriate threshold could alleviate clinician burden.

The objective of the present study was to estimate the algorithm performance of the next generation leadless pacemaker, Micra AV2, and compare the estimated algorithm performance to that of Micra AV. The study estimated the expected reduction in programming burden after implant, the associated clinical time savings, and the improvement in AVS at high sinus rates (80–100 bpm). It was hypothesized that the mean AVS following the Atrial Sensing Setup algorithm would be significantly greater in Micra AV2 as compared to Micra AV. Similarly, the mean AVS in the 80–100 bpm range was hypothesized to be significantly greater when using the Auto + A3 Threshold algorithm in Micra AV2 compared to the Auto A3 Threshold algorithm in Micra AV.

## Methods

### AVS Algorithm Updates

**Fig. 3:**
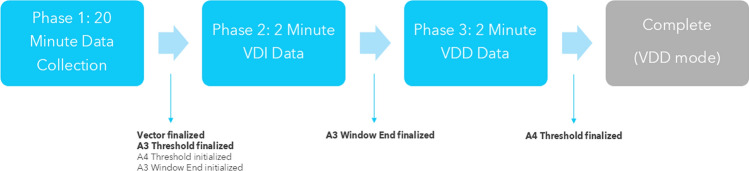
Accelerometer vector selection and A3 Thresholds are finalized in the first phase of Atrial Sensing Setup. The A3 Window End parameter is finalized in the second phase. The A4 Threshold is finalized in the third phase

The first algorithm update is to the device’s Atrial Sensing Setup feature. The Atrial Sensing Setup algorithm’s primary update in Micra AV2 was to improve the A3 Window End setting. The first generation Atrial Sensing Setup often selects an A3 Window End value that is too long, which makes it difficult to achieve proper atrial contraction detection and AVS. For context, the Atrial Sensing Setup algorithm is a 24-min test that primarily operates in VDI mode. The Atrial Sensing Setup algorithm chooses the accelerometer vector combination, A3 Threshold, A3 Window End, and A4 Threshold. The first 20 min of the algorithm include the collection of A3, A4, and A3 Window End data for different combinations of the individual vectors that make up the 3-axis accelerometer (Vector 1, Vector 2, and Vector 3). The four accelerometer vector combinations evaluated during the Atrial Sensing Setup algorithm execution are: Vector 1 + Vector 2 (1 + 2), Vector 1 + Vector 3 (1 + 3), Vector 2 + Vector 3 (2 + 3), and Vector 1 + Vector 2 + Vector 3 (1 + 2 + 3). The two-vector combination with the largest A4 signal is chosen, and the vector combination 1 + 2 + 3 is chosen in the event where none of the two-vector combinations have large A4 signals. The A3 Threshold is finalized, and the A3 Window End and A4 Threshold are initialized (Fig. 3). The second phase is a 2-min VDI phase where the A3 Window End is adjusted. The A3 Window End is adjusted by detecting the time of the last A3 signal that was close in amplitude of the A4 Threshold. Therefore, a small A4 Threshold will tend to extend the A3 Window End and a large A4 Threshold will tend to shorten the A3 Window End. In the Micra AV device, the A4 Threshold during this 2-min VDI phase was fixed at 1.2 m/s^2^, but in the Micra AV2 device, the A4 Threshold is based on the initialized A4 Threshold from the first phase of the algorithm. In general, shorter A3 Window End values will be expected from this method. The third phase adjusts the A4 Threshold based on the fine-tuned A3 Window End and was not modified between the two devices.

The second algorithm update is to the A3 Threshold algorithm. The Micra AV Auto A3 Threshold algorithm adjusted the A3 Threshold based on both the A4 Threshold value and the A3 signal amplitude. Because the A3 signal amplitude was used in the A3 Threshold adjustment, extended periods of high sinus rates (typically greater than 80 bpm) led to a higher signal in the A3 window being measured, because the device was measuring the A3 + A4 (A7) signal. This caused the A3 Threshold to automatically adjust higher until undersensing of the A7 signal occurred. Figure 2 shows an example of the A3 signal amplitude over 24 h and the Micra AV Auto A3 Threshold algorithm. Very large maximum A3 amplitudes reflect periods of higher sinus rates when the A4 signal superimposes on the A3 Threshold (A3 + A4). The changes in how the A3 Threshold is set in Micra AV2 were also incorporated into the updated Atrial Sensing Setup algorithm.

**Fig. 4:**
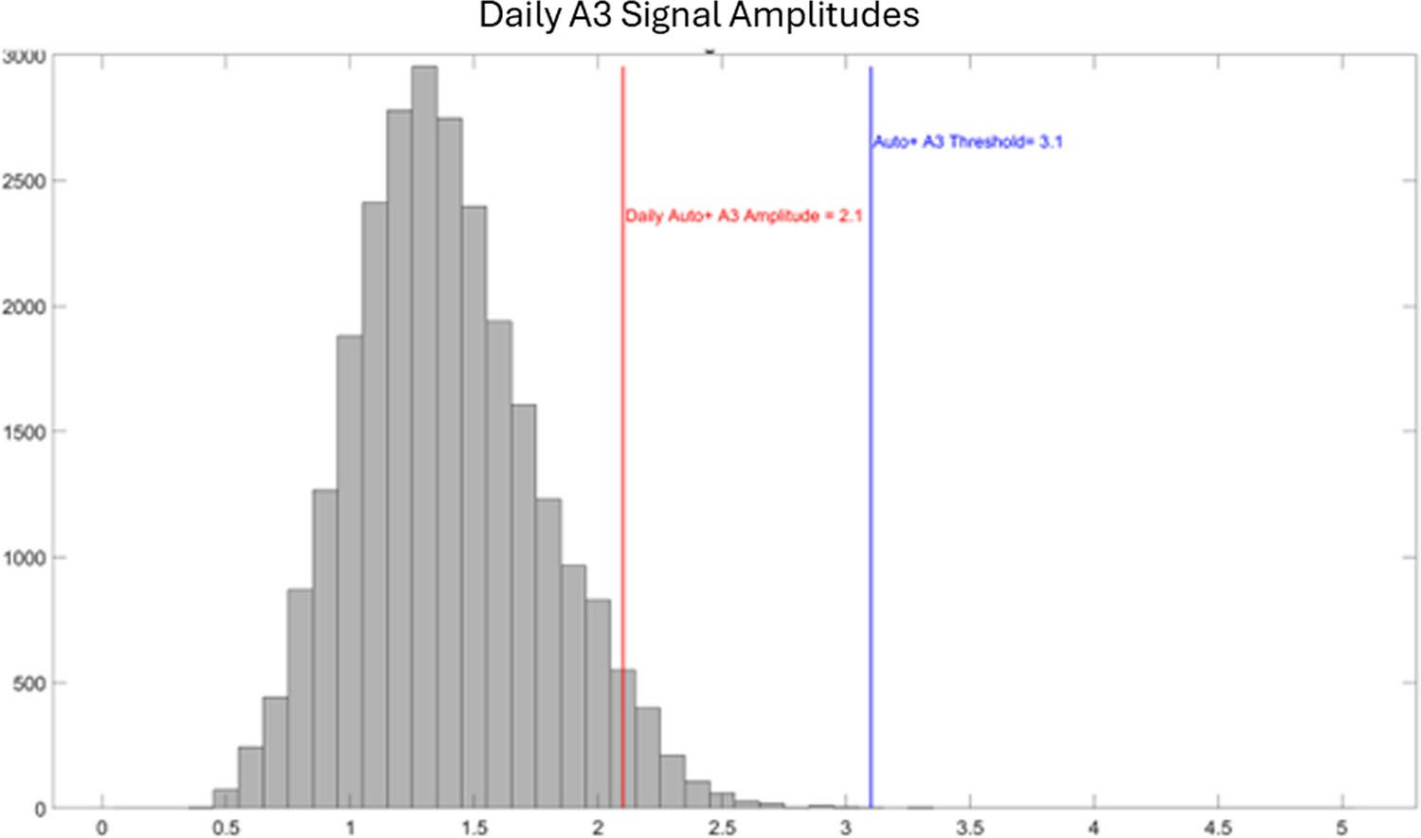
Histogram of A3 signal amplitudes used for the Micra AV2 Auto + A3 Threshold algorithm. After this histogram is collected, the 95th percentile is used to determine the daily A3 amplitude that should not be detected by the Micra AV2 device. A programmable amplitude margin can then be applied (nominal = 1.0 m/s^2^) to further help avoid A3 oversensing

**Fig. 5:**
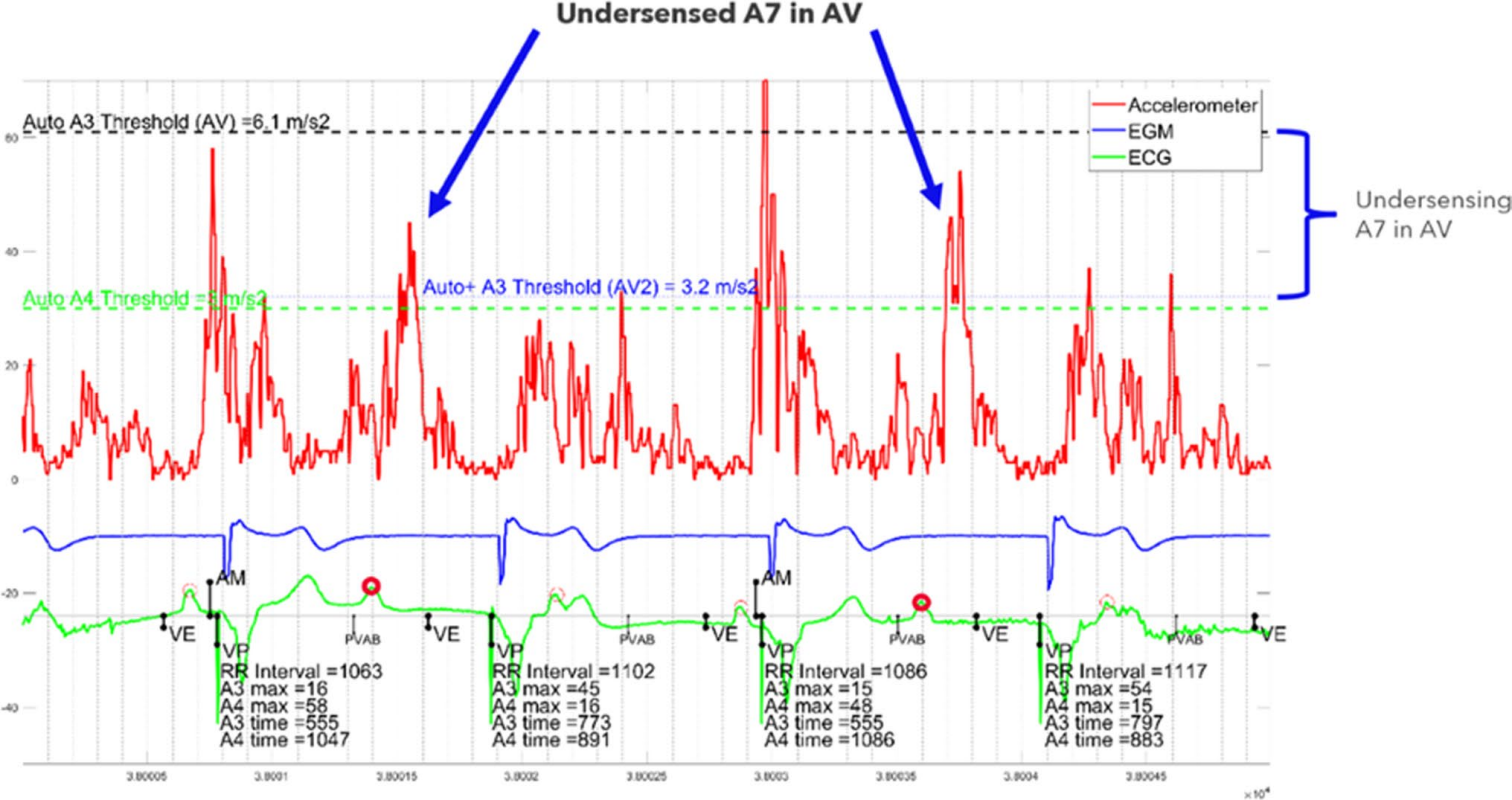
A 5-s strip at 10.55 h from the 24-h recording of an AccelAV patient. The accelerometer signal is plotted in red, the EGM in blue, and the ECG in green. The Micra AV Auto A3 Threshold was set to 3.2 m/s^2^. Two A7s (A4 occurring in the A3 window) are shown. The measured A3 amplitude of 4.5 and 5.4 m/s^2^ are undersensed with the Micra AV Auto A3 Threshold, but are detected with the Micra AV2 Auto + A3 Threshold

The goal of the Micra AV2 algorithm, Auto + A3 Threshold, is to set the A3 Threshold above the baseline A3 signal to guard against A3 oversensing, but low enough to detect the summated A3 + A4 (A7) signal. Auto + A3 Threshold finds cycles that have isolated A3 signals and not A3 + A4 (A7) signals and stores those values in an A3 Threshold histogram (Fig. 4). Once per day, the A3 Threshold histogram is analyzed, and a Daily A3 amplitude is determined. A programmable A3 AmplitudeMargin (nominal = 1.0 m/s^2^) is applied to create the Micra AV2 Auto + A3 Threshold. An example of A3 undersensing observed with the high Micra AV Auto A3 threshold algorithm in the 5-s strip at 10.55 h is shown in Fig. 5. The Micra AV2 Auto + A3 Threshold appropriately detects the A7 signals.

### AVS Algorithm Performance Modeling

The AccelAV clinical study collected accelerometer data at implant, 1-month, and 3-month follow-ups. A specialized Holter monitor capable of storing accelerometer waveforms, electrograms (EGMs), device markers, and electrocardiogram (ECG) data was placed for the duration of the study procedures. It stored the amplitude and timing of the A3 and A4 accelerometer signals. These accelerometer measurements were collected over time to construct distributions of the A3 and A4 signals for each patient at implant and at 1-month. These A3 and A4 signal distributions coupled with average sinus rate intervals and premature atrial and ventricular contraction (PAC/PVC) burden in the ambulatory setting enabled virtual patients to be constructed with similar characteristics to the AccelAV patients.

**Fig. 6:**
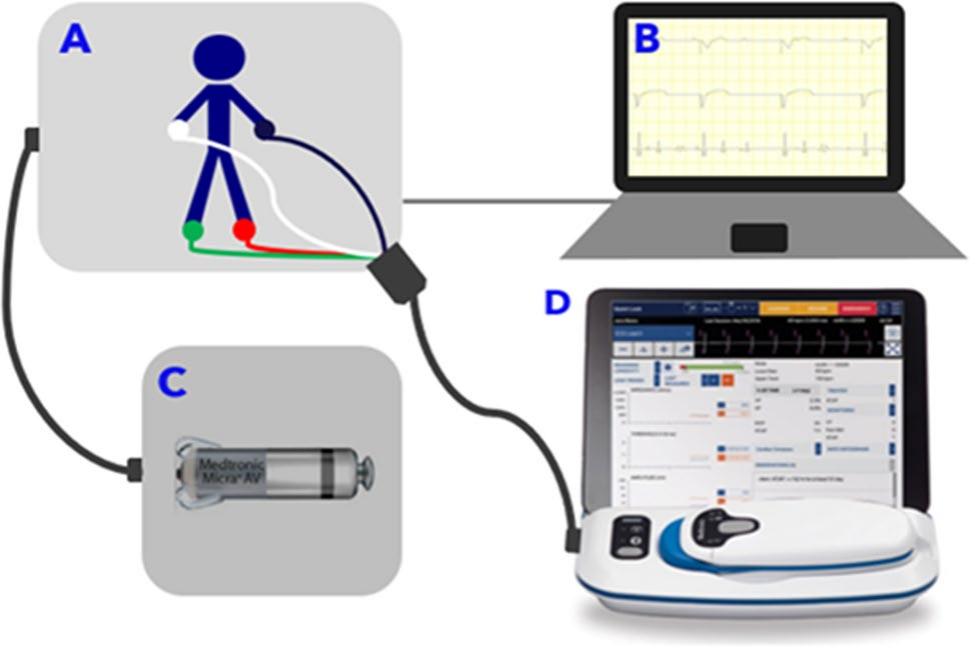
Bench testing setup consisting of the InterSim III Interface simulator box (**a**), laptop with InterSim software (**b**), AV/AV2 hybrid adaptor box (**c**), and CareLink SmartSyncTM programmer (**d**). The InterSim interface simulator box is connected to the programmer via ECG cables

A heart simulator (InterSim III Interface, Germany, www.intersim3.com) was developed that constructs realistic and tunable accelerometer signals, intracardiac electrogram, and surface ECG and can interface with the Micra AV or AV2 device to test the device algorithms. The simulation setup consists of a heart simulator interface box, AV/AV2 hybrid adaptor box, laptop with InterSim III software, and device programmer (Fig. 6).

**Fig. 7:**
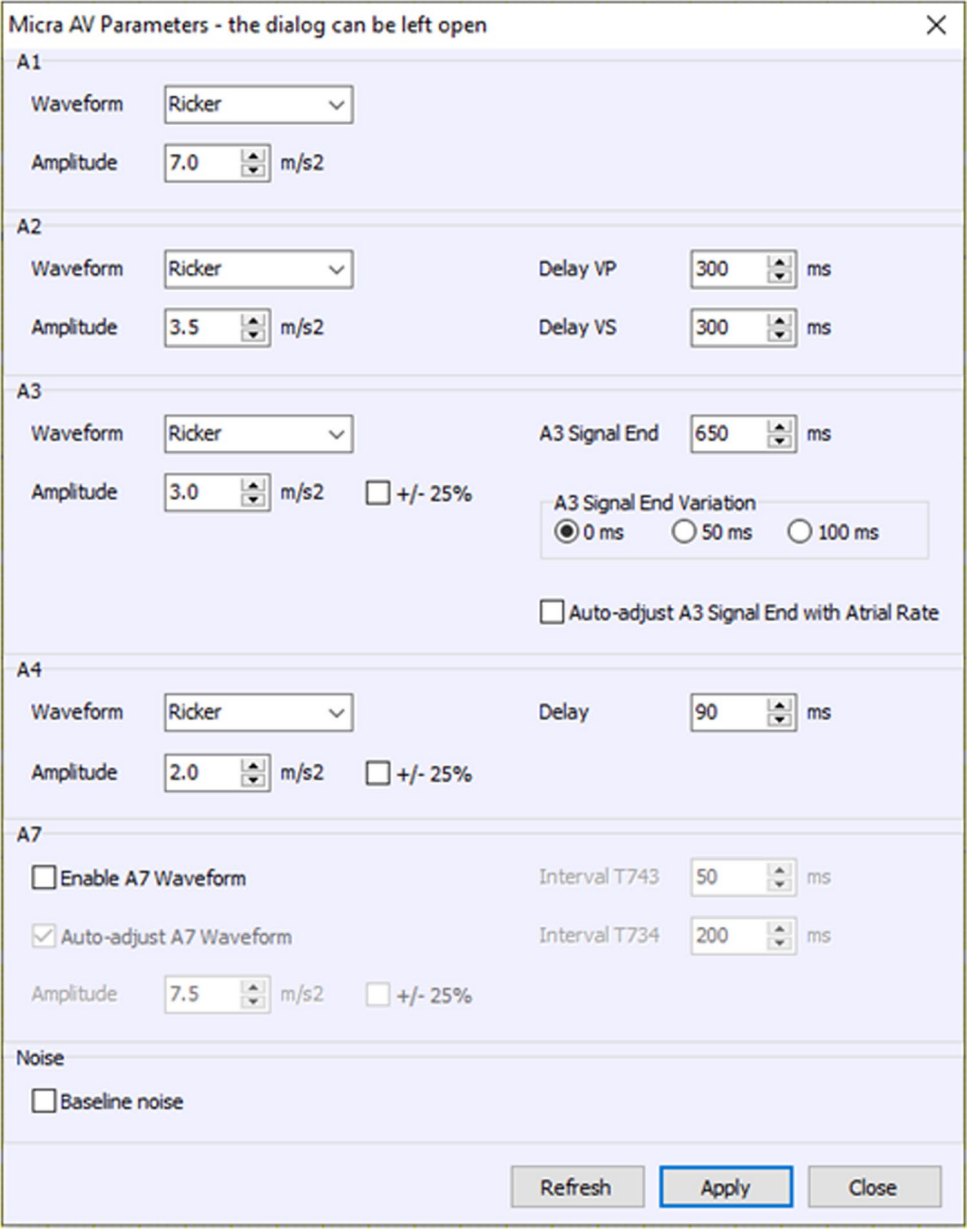
Programmable parameters for constructing the accelerometer signal in InterSim III Interface. The Ricker waveform (Eq. 1) was used for all signals in the simulations. The amplitudes and timings of the A1, A2, A3, A4, and A7 signals can be changed using the InterSimIII software. The A2 Delay represents the time between the QRS on the ECG and the start of the A2 signal. The timing can be different following a VP (ventricular pace) or VS (ventricular sense). The default for both is 300 ms. The A3 Signal End timing can be adjusted as desired and the A3 Signal End Variation randomly varies the A3 Signal End. The random value results from a Gaussian Distribution (standard deviation is 17ms for the 50 ms option and 33 ms for the 100 ms option). The Auto-adjust A3 Signal End with Atrial Option adjusts the A3 Signal End with the Atrial Rate when rates are greater than 60 bpm. The A4 Delay represents the timing between the start of the P-wave and the start of the A4 signal. The default is 90ms. Baseline noise can be added to the signal but was not utilized in these presented simulations

The AV/AV2 hybrid boxes use modified Micra AV and AV2 devices that allow the accelerometer signal to be input electrically into the device from the InterSim III Interface to enable simulation of the accelerometer signals without the need for a mechanical shaker table. The laptop with InterSim III Interface software allows control of patient sinus rate intervals as well as accelerometer signal component parameters including amplitude and timing (Fig. 7).

The InterSim III software includes support for a macro/scripting language that enabled a Monte Carlo simulation to be developed and executed. The Monte Carlo simulation varies the P-wave/A4 timing (sinus rate interval), A4 amplitude, and A3 amplitude within each patient’s signal distribution on a beat-by-beat basis. As mentioned previously, the sinus rate interval, A4 amplitudes, and A3 amplitudes were extracted from AccelAV patients, so the range of these parameter values varied based on the specific patient dataset from which they were extracted. The sinus rate interval was modulated with + /− 5% random variability around the average. The simulated A4 and A3 amplitudes matched the ranges and normal distributions of the A4 and A3 amplitudes of the patient dataset from which they were extracted. The software also included support for delivering summated A7 signals. In the real world, the degree of overlap of the A3 and A4 signals can impact the A7 summation, along with other physiologic factors. The generation of the A7 signal in the software is simplified by generating an A7 whenever the A3 and A4 signals overlap. The accelerometer signal morphology was modeled as a Ricker wavelet (1):

1 $$\left[1- \left(2*{\uppi }^{2}*{f}^{2}*{t}^{2}\right)\right]*{\text{e}}^{{-\uppi }^{2}*{f}^{2}*{t}^{2}}$$

*f* = 20 Hz (peak frequency) *t* = time (ms)

**Fig. 8:**
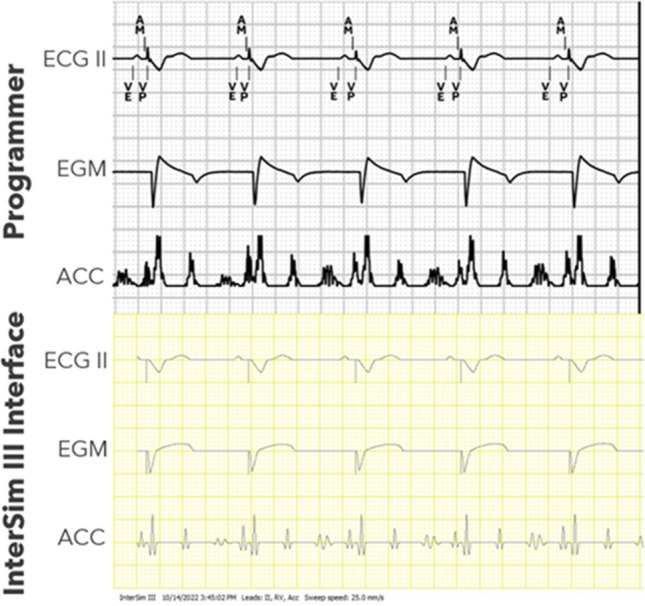
Simulated ECG, EGM, and accelerometer signals recorded simultaneously through CareLink SmartSyncTM programmer (top panel) and InterSim III software (bottom panel). The programmer’s accelerometer waveform is filtered and rectified, while the InterSim III accelerometer waveform is raw

Fig. 8 provides a closer look at the cardiac waveforms displayed simultaneously on the InterSim III Interface software and on the Micra AV and AV2 device programmer.

**Fig. 9:**
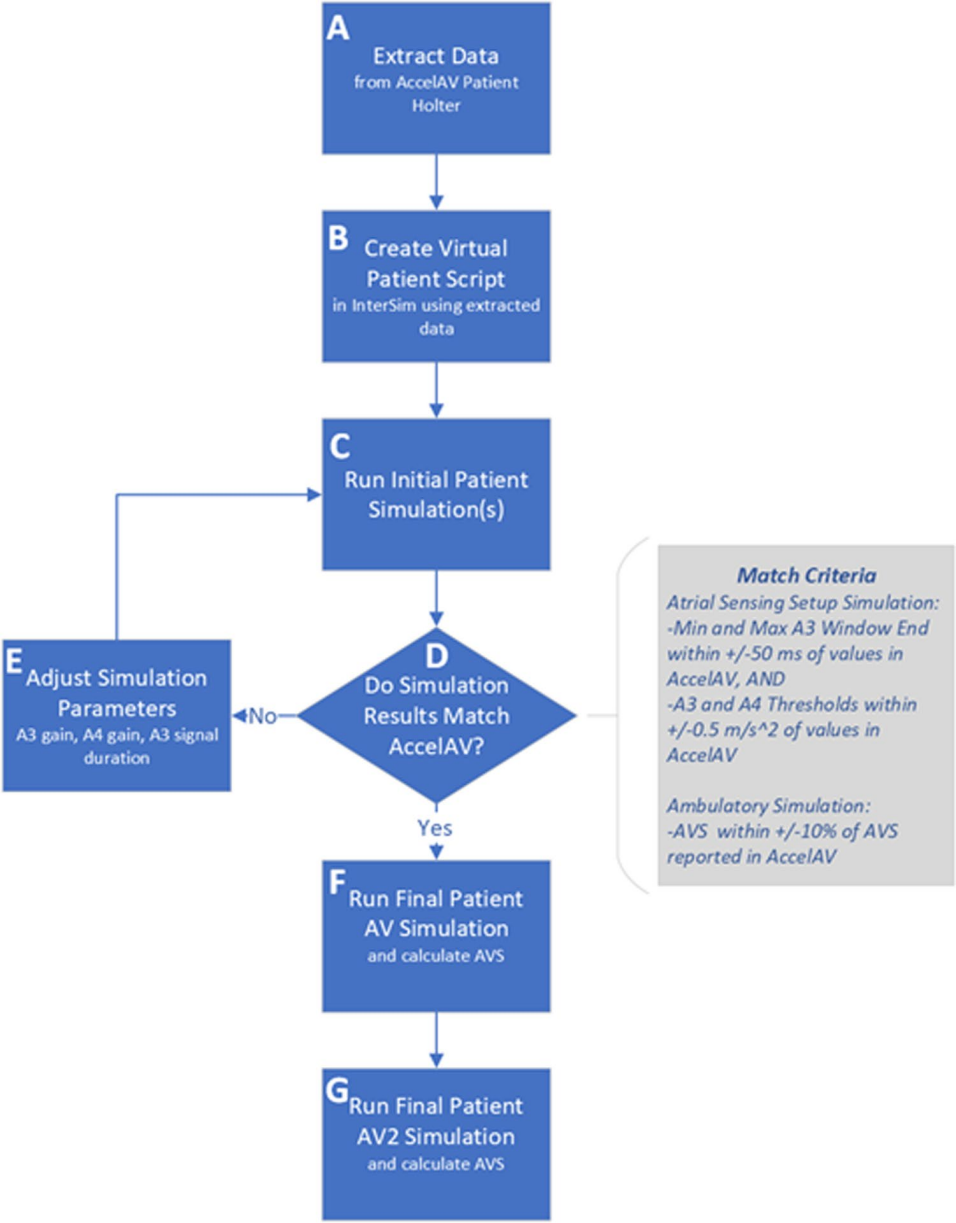
High-level process flow for creating virtual patients and using them to assess updated device algorithm performance

Using A3 and A4 distributions, sinus rate intervals, and PAC/PVC burden extracted from AccelAV patients as inputs, the InterSim III simulator allowed for the testing of different device algorithms in virtual patients. The sinus rate intervals were modeled based on the patient’s average hourly sinus rate intervals with up to 10% random variability (+/− 5%). The same average sinus rate intervals were used for both the Micra AV and Micra AV2 simulations, but the variability could have differed as it was random. The PAC/PVC burden was modeled for the ambulatory simulations only. The A1 and A2 signal amplitudes and timings were not extracted from the AccelAV patient data; instead, the A1 and A2 signal sizes and timings were fixed. The A1 and A2 signals are blanked using Post Ventricular Atrial Blanking (PVAB), and the goal of this modeling was not to test Micra AV2’s Auto PVAB feature, but to isolate and test the A3 Threshold features. Figure 9 shows the high-level process followed to assess device algorithm performance using virtual patients. To be consistent with Medtronic AVS calculations in other studies [2, 3], cardiac cycles were defined as synchronous if a ventricular marker followed the P-wave by ≤ 300 ms.

### Atrial Sensing Setup Simulation

The AccelAV implant Holter recordings from patients with complete AV block, normal sinus function, and good telemetry were selected (*n* = 30) and used to simulate the Atrial Sensing Setup updates (Fig. 9). The accelerometer measurements in the chosen accelerometer vector combination from these recordings were stored as A3 and A4 amplitude distributions, along with the average sinus rate interval during the setup test, to create a virtual patient script for each AccelAV patient (Fig. 9b). The Monte Carlo virtual patient simulation was then run via the patient script in InterSim III Interface while the Atrial Sensing Setup algorithm was executed on the Micra AV device (9(c)). The parameters chosen during the simulation were compared to the actual AccelAV study chosen parameters to confirm the virtual patient accurately modeled the AccelAV patient (Fig. 9d). In cases where the simulation did not match the AccelAV patient Atrial Sensing Setup results, slight gain adjustments of the A3 or A4 signal distributions or adjustment of the A3 signal duration were performed (Fig. 9e). When the parameters chosen by the AccelAV and Micra AV simulation matched within + /- 50 ms for Min and Max A3 Window End and + /− 0.5 m/s^2^ for A3 and A4 Thresholds, that individual patient script was considered complete. The finalized virtual patient was then simulated for 30 min after the Atrial Sensing Setup algorithm had completed on both Micra AV and AV2 devices (Fig. 9f and g). The detection parameters chosen were compared and the subsequent AVS from simulations using the chosen detection parameters was measured and compared between the two devices (Table 1).

**Table 1:** Programmed parameters by the atrial sensing setup algorithm

*n* = 30	AccelAV	AV simulation	AV2 simulation
Min A3 Window End (ms)	775 ± 25	775 ± 50	700 ± 50
Max A3 Window End (ms)	900 ± 50	900 ± 50	800 ± 50
A3 Threshold (m/s^2^)	5.0 ± 1.6	5.0 ± 1.4	3.4 ± 1.2
A4 Threshold (m/s^2^)	1.8 ± 0.6	2.0 ± 0.6	2.2 ± 0.6

The AccelAV column is the averaged parameters from 30 patients in the AccelAV trial. The Micra AV simulation matches these parameters. The Micra AV2 simulation demonstrates the improved Atrial Sensing Setup algorithm by selecting shorter A3 window ends and lower A3 thresholds

### Automated A3 Threshold Algorithm Simulation

Ambulatory (up to 24-h) Holter recordings were collected at the 1-month post-implant AccelAV follow-up. Twenty Holters from patients with complete AV block, normal sinus function, and good telemetry were used to simulate the A3 Threshold algorithm updates (Fig. 9). The accelerometer measurements were stored in A3 and A4 distributions along with the hourly average sinus rate interval and percentage of beats that were PACs or PVCs. These data were used to construct the initial virtual patient scripts (Fig. 9b). Each initial virtual patient script was then run in InterSim III Interface, with the connected Micra AV device programming matching the AccelAV 1-month programming (Fig. 9c). The resulting AVS values between the simulation and AccelAV were compared (Fig. 9d). When the simulation and AccelAV AVS results diverged, the AccelAV Holter was further reviewed to understand the causes of loss of AVS. The two main causes of loss of AVS were: (1) undersensing of the A7 signal at higher sinus rates (typically greater than 80bpm) and (2) oversensing of the A3 signal when a short A3 Window End and low fixed A4 Threshold were programmed. Gain adjustments to the A3/A4 amplitude distributions and/or A3 signal duration were made to mimic the loss of AVS scenarios in the patients where they occurred (Fig. 9e). When the AVS from the simulation matched within + /− 10% of the AVS achieved in the AccelAV study, that individual patient script was considered complete and ready for final simulation. Two simulations were conducted with A3 Threshold algorithms enabled in both the Micra AV and AV2 devices (Fig. 9f and g, respectively).

The Micra AV2 Auto + A3 Threshold for each patient was determined from the AccelAV Holter recording using a computer simulation of the new Micra AV2 Auto + A3 Threshold algorithm. Nominal detection parameter settings were used for both simulations, except for shorter A3 Window End values in the 700–800 ms range. The Post Ventricular Atrial Blanking and Upper Tracking Rate were left at their nominal values (550 ms, 105 bpm). These parameter settings enabled strict comparison of the two A3 Threshold algorithms. The resulting AVS values from these two simulations were compared for each patient across all sinus rates as well as for rates between the A3 window range and Upper Tracking Rate (i.e., 80–100 bpm).

### Real-World Experience Survey

To understand how the improvements to Atrial Sensing Setup impacted clinic time savings, two surveys were conducted. The first survey collected data on Micra AV and was conducted shortly after the Micra AV launch, and the second survey collected data on Micra AV2 and was conducted shortly after the Micra AV2 launch. Each survey collected data about the estimated time to complete the device setup at the pre-hospital discharge device check, which is the device check done after Atrial Sensing Setup has run and before the patient leaves the hospital. Each survey had the same predefined time ranges for selection which included less than 5, 5–10, 11–20 min, and greater than 20 min. Both surveys were sent to U.S. Medtronic field representatives who were responsible for programming the devices. Each field representative was instructed to fill out a survey after each of their first few patients. Thus, each survey response represented results from a single patient. The survey was anonymous, and participation was optional. After approximately 100 responses were received for each survey, the surveys were closed.

### Statistical Methods

Based on the previous Micra clinical studies, MARVEL 2 and AccelAV [2, 3], it was assumed 50% of virtual patients would have > 70% AVS following the original Micra AV Atrial Sensing Setup algorithm. Assuming that the Micra AV2 Atrial Sensing Setup algorithm would increase the likelihood of achieving > 70 to 90%, it was determined that 30 virtual patients would provide 90% power to detect a difference in the proportion of patients with > 70% AVS following Atrial Sensing Setup between the Micra AV and AV2 Atrial Sensing Setup algorithms.

McNemar’s test was used to compare the proportion of virtual patients with > 70% AVS following Atrial Sensing Setup between the Micra AV and AV2 algorithms. Additionally, logistic regression models using generalized estimating equations were used to compare AVS by algorithm during the 30-min period following the completion of the Atrial Sensing Setup algorithm and within sinus rate during the ambulatory simulation period. P-values < 0.05 were considered statistically significant. SAS v9.4 (Cary, NC) or R v4.2.1 (R Foundation for Statistical Computing). Survey results were reported as absolute percentages and summary statistics were obtained using medians with basic formulas in Excel.

## Results

**Fig. 10:**
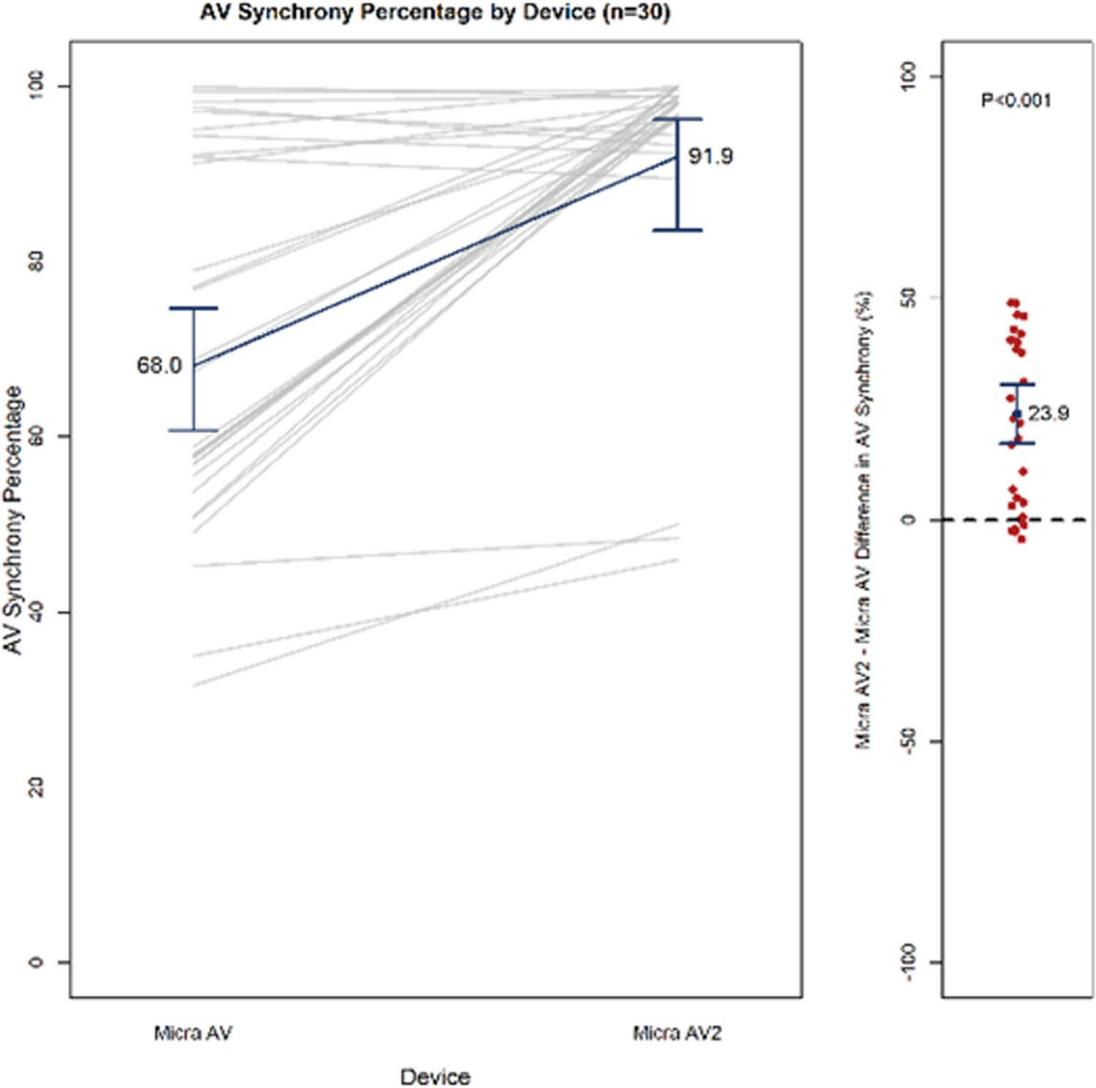
AVS achieved during the 30-min simulation immediately following the completion of the Atrial Sensing Setup algorithm. Left: AVS with Micra AV and AV2. Gray lines indicate individual patient values. Blue line connects averages. Right: Change in AVS with Micra AV2 vs. AV. Red circles are individual patients; blue circle is the average change. Error bars are 95% confidence intervals

Atrial Sensing Setup was simulated on 30 virtual patients. Figure 10 shows the AVS achieved during the 30-min simulation immediately following the completion of the Atrial Sensing Setup algorithm with Micra AV2. Mean AVS with Micra AV was 68.0% (95% CI 60.7–74.6%) vs. 91.9% (95% CI 83.5–96.2%) with Micra AV2 during the 30-min resting period following the completion of the Atrial Sensing Setup algorithm (*p* < 0.001). Thirteen of 30 (43%) patients achieved greater than 70% AVS with Micra AV, while 27 of 30 patients (90%) had greater than 70% with Micra AV2 (*p* < 0.001). The parameters chosen by the algorithm are shown in Table 1. When comparing outputs of Micra AV and AV2 Atrial Sensing Setup algorithms, shorter A3 Window End times were seen in 29 of 30 virtual patients. The shorter A3 Window End and lower A3 Threshold selected by the Micra AV2 Atrial Sensing Setup were the key contributors to the improved AVS observed.

**Fig. 11:**
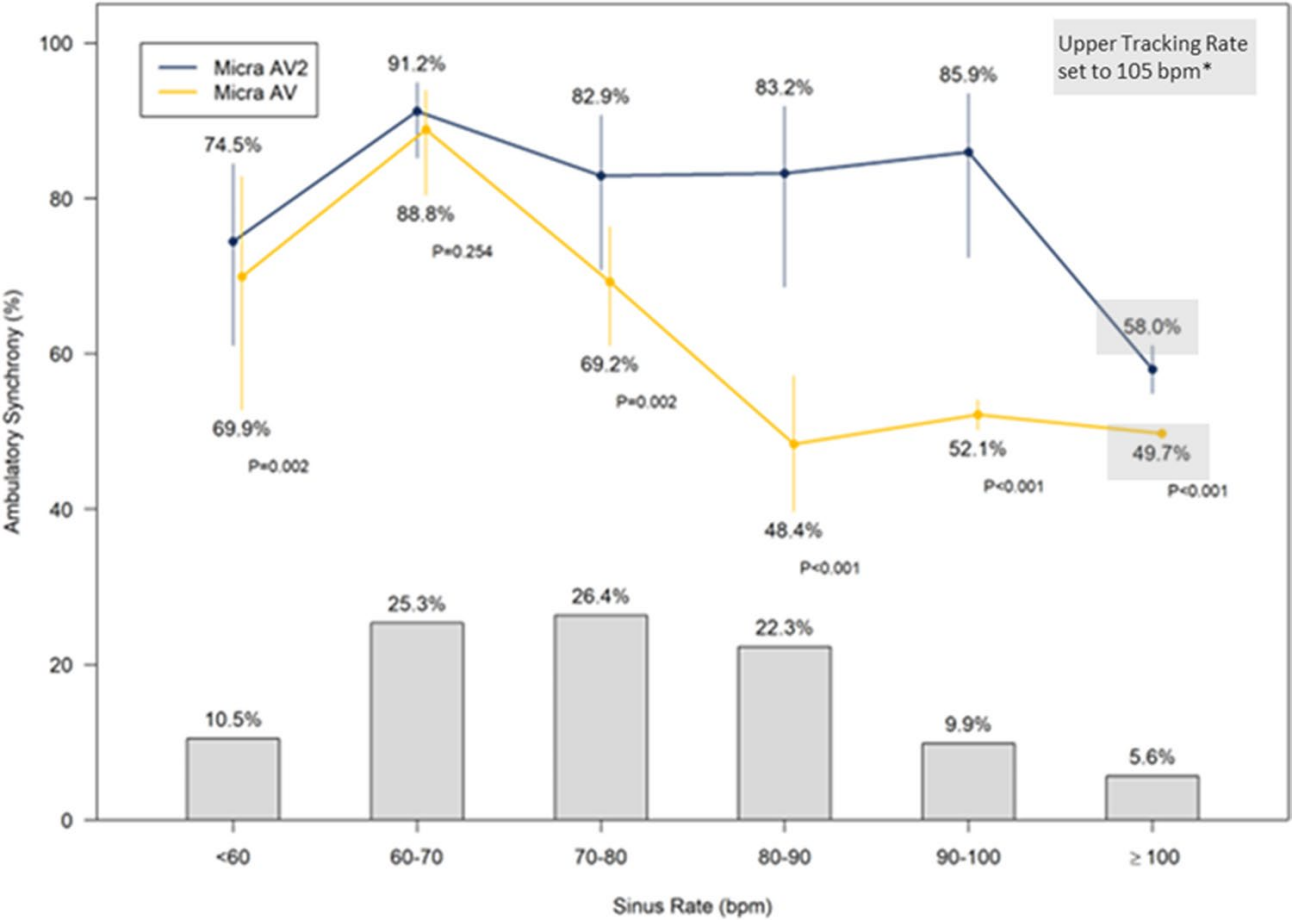
Ambulatory AVS measured during simulations. Lines connect average AVS by sinus rate. Error bars represent 95% confidence intervals. Gray bars indicate the percentage of total cardiac cycles within each sinus rate category. *Upper Tracking Rate was programmed to 105 bpm for both devices; capability of delivering AV synchronous pacing beyond 105 bpm was not simulated

In addition, the virtual patients created for the ambulatory simulations were determined to match the AccelAV patients well; the mean and standard deviation AVS for the 20 patients in AccelAV was 75.5 ± 14.5%, while the mean and standard deviation AVS for the 20 virtual patients when programmed with AccelAV parameters was 76.2 ± 17.5%. Figure 11 shows the ambulatory AVS measured during the A3 Threshold algorithm simulations by sinus rate. When directly comparing the Micra AV2 Auto + A3 Threshold algorithm and the Micra AV Auto A3 Threshold algorithm (A3 Window End 700–800ms, other parameters nominal), mean AVS was 66.8% (95% CI 59.1–73.7%) with Micra AV compared to 83.1% (95% CI 75.2–88.8%) with Micra AV2 in the simulations (*p* < 0.001). AVS in the 80–100 bpm range was 49.5% (95% CI 43.2–55.8%) with Micra AV and increased to 84.1% (95% CI 71.3–91.8%) with Micra AV2 (*p* < 0.001).

During the simulations, the mean Micra AV2 Auto + A3 Threshold was 3.4 vs. 4.0 m/s^2^ for the Micra AV Auto A3 Threshold. The A3 Threshold was likely to increase during the simulation due to the frequent A3 Threshold updates that occur in the Micra AV Auto A3 Threshold algorithm. In contrast, since the Micra AV2 Auto + A3 Threshold algorithm updates only once daily, it is stable during periods of higher heart rates. The lower A3 Threshold was the key factor in the improvement in AVS at higher heart rates (80–100 bpm).

**Fig. 12:**
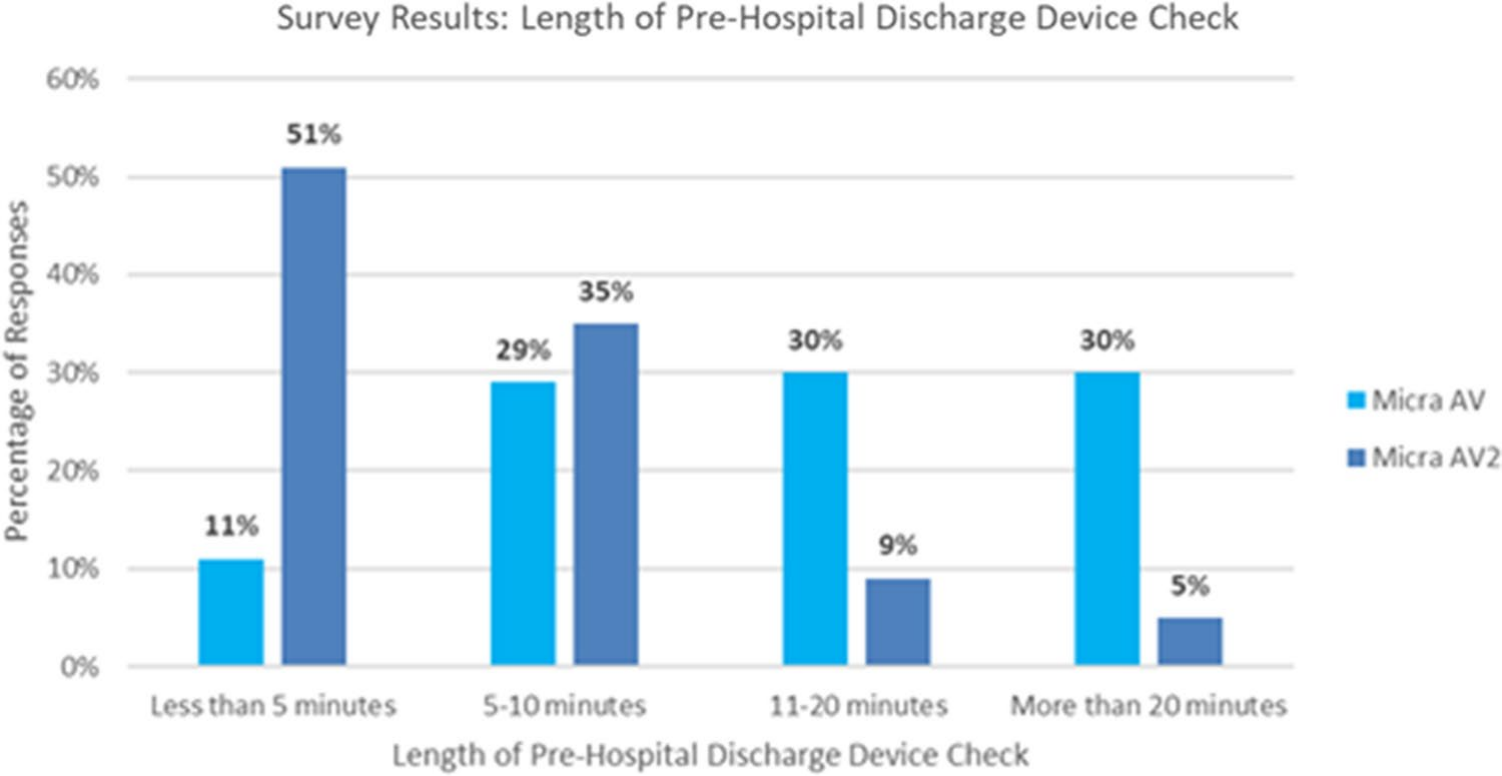
Length of pre-hospital discharge device check from survey results. Results shown in percentage. Total Micra AV survey responses were 104. Total Micra AV2 survey responses were 98

The real-world, pre-hospital discharge surveys received 104 responses for Micra AV and 98 responses for Micra AV2. Out of those responses, 91% of Micra AV and 84% of Micra AV2 respondents had completed 10 or fewer Micra AV or Micra AV2 device checks, respectively. Figure 12 shows the estimated time it took to complete the pre-hospital discharge checks for each device type. For Micra AV, the time it took to complete the pre-hospital discharge device check was split evenly (29, 30, and 30%) among the longer response options with only 11% of responses in the shortest option of less than 5 min. Less than half (40%) of device checks took 10 min or less. For Micra AV2, the responses are skewed towards the minimum response options, with the majority (51%) of checks taking less than 5 min and 86% of device checks taking 10 min or less. The median device check time for Micra AV was 11–20 min whereas the median device check time for Micra AV2 was less than 5 min. Assuming the median of the response range represents the average device check time, Micra AV2’s median device check time is 13 min less than that of Micra AV (2.5 vs. 15.5 min). Using the median time savings of 13 min, an estimated 21.7 h of clinic time would be saved per 100 implants. Additionally, the time it took to check Micra AV2 was less variable, with 86% of checks taking 10 min or less. Micra AV had only 40% of device checks taking 10 min or less and 30% taking more than 20 min.

## Discussion

The advent and widespread clinical application of leadless cardiac pacing technology has been a pivotal landmark in the field of clinical cardiac electrophysiology within the past decade. The first leadless pacemakers available offered ventricular only sensing and pacing (VVI mode), while a second generation of leadless pacemakers added atrial sensing capabilities (VDD mode) to the same device implanted in the right ventricle using an embedded accelerometer to detect the atrial mechanical contraction.

As with any new technology, the initial clinical application of VDD leadless pacemakers has been characterized by a learning curve among users. Specifically, the novel algorithms designed to enable AVS pacing in the Micra AV device have received attention. While mechanical sensing as a modality of sensing atrial activity is unlikely to be perfect, early clinical experience with Micra AV suggests that the ability of these novel algorithms to deliver AVS under nominal settings can be partially compromised in some scenarios. In the AccelAV clinical study of Micra AV, it was observed that some patients experienced suboptimal AVS due to inadequate device parameter programming. The Optimize substudy looked at a subset of patients (*n* = 20) who underwent programming changes to improve A4 signal recognition and observed an increase in median ambulatory AVS percentage from 71.9 to 82.6% [2].

This early clinical experience has led to a better understanding and subsequent optimization of the device engineering and post-implant programming. In addition to updating programming recommendations to improve AVS with Micra AV, Medtronic made further algorithm updates in Micra AV2 with the goal of automatically improving AVS while reducing manual programming burden. The present analyses sought to assess the performance impact of these algorithm updates in a simulated environment with supplemental real-world analysis.

The first improvement geared towards better recognition and differentiation of the A3 and A4 signals was the implementation of the Micra AV2 Atrial Sensing Setup algorithm, which resulted in shortening of the A3 window in 29 of the 30 virtual patients analyzed by this study. This is an important confirmation of the clinical observation that the originally programmed A3 windows were often long enough to sometimes inadvertently include the A4 signal, therefore causing atrial contraction undersensing and inadequate AVS timing. After enabling the enhanced Atrial Sensing Setup algorithm, the proportion of virtual patients achieving at least 70% AVS was as high as 90% during the 30 min following the algorithm-directed programming of the A3 detection window.

The A3 Threshold algorithm updates were also assessed in this virtual patient study. In Micra AV, the Auto A3 Threshold algorithm did not appropriately differentiate the isolated A3 signal from the superimposed A3 and A4 signals, which occurred in faster heart rates. The adoption of an improved Auto + A3 Threshold algorithm, which assesses the size of the A3 signal in scenarios when the summation phenomenon is not present, was responsible for better AVS rates, particularly during fast heart rates (80–100 bpm), which have been associated with lower AVS percentages due to the physiological changes associated with increased chronotropic response.

The virtual patient model adopted in this study, which uses data from clinical trial patients implanted with the Micra AV leadless pacemaker, has been utilized in the past for predicting the impact of technological improvements associated with the other implantable cardiac devices [7]. This is certainly not different in the field of leadless cardiac pacing, and the present study indicates automatic algorithm and programming improvements are expected to occur with significantly favorable clinical consequences to the appropriately selected patient population.

Although the virtual study was useful to predict programming improvements, model limitations prevented the ability to determine the real-world time savings acquired by the Atrial Sensing Setup enhancements via the simulated model. Instead, real-world surveys of end users’ experiences with the pre-hospital discharge device check after either a Micra AV or a Micra AV2 implant was used. The surveys showed the median estimated time spent checking a Micra AV2 device was 13 min shorter than the median estimated time spent checking a Micra AV device, with the majority of Micra AV2 device checks taking less than 5 min. Not only was the time checking the Micra AV2 device shorter, but it was also more predictable with less variability encountered in terms of device programming. Though clinical time saved is admittedly a challenging variable to quantity, it was assumed that having shorter, more predictable device checks may aid in clinic staffing and scheduling. This might also mean that patients can be discharged sooner, knowing what to expect in terms of how long their routine device checks will take. These survey results also support that the reduction in need for manual programming predicted by the simulated model is occurring in the real-world.

There are several limitations to the simulation analyses. While variation in simulation parameter values was introduced when feasible, not all physiologic variation could be accounted for. For example, the simulation draws parameters for each patient’s distributions on a beat-to-beat basis assuming that the parameters for each beat parameter are independent. In reality, there is a relationship between these parameters due to electromechanical coupling, but for simplicity these parameters were utilized as if independent in the presented simulations. Also, simplified variables of the simulations are discussed in the methods section and include A1 and A2 amplitude and timing, A3 and A4 waveform, PAC/PVCs, and heart rate variation. Specifically, the A7 summation was implemented as a binary process in the simulation, meaning a summated waveform was output by InterSim III when the A3 and A4 signals overlapped. In the real-world, the degree of summation between the two signals depends upon a variety of physiologic parameters that are difficult to characterize for simulation. This binary summation may contribute to the strong performance of the AV2 Atrial Sensing Setup algorithm simulation in the 80–100 bpm range. Due to the limited amount of clinical data, the Micra AV2 Auto + A3 Threshold was determined from data that was re-utilized for simulation, which could introduce bias. Although the authors are confident the simulation results demonstrate the relative algorithm performance improvements, the absolute AVS improvements resulting from the simulations should be interpreted with caution due to the limitations outlined here.

The real-world survey limitations are that the survey respondents only included Medtronic field representatives in the United States. Although steps were taken to prevent bias, like making the survey results anonymous and requesting surveys be completed following each case, there are other device users missing from the survey respondents, like device nurses. A categorical survey was selected to gain clinical insights without the need for a costly formal clinical study. Though both groups had a similar amount of experience with the product they were being surveyed on, some learnings from completing Micra AV device checks over time could impact the speed at which respondents were able to complete Micra AV2 device checks.

## Conclusion

Modeling and simulations performed to assess the performance of the Micra AV2 leadless pacemaker show an increase in the number of patients with > 70% AVS without the need for manual programming and significant automatic AVS improvements in sinus rates greater than 80 bpm when compared to Micra AV. Additionally, survey results show the reduction in manual programming has led to shorter and more predictable Micra AV2 device checks in the real world.

## Data Availability

The data used to perform the analyses in this paper will not be made publicly available.

## References

[1] Chinitz, L., et al. Accelerometer-based atrioventricular synchronous pacing with a ventricular leadless pacemaker: Results from the Micra atrioventricular feasibility studies. *Heart Rhythm*. 15(9):1363–1371, 2018. 10.1016/j.hrthm.2018.05.004. 29758405 10.1016/j.hrthm.2018.05.004

[2] Chinitz, L. A., et al. Ambulatory atrioventricular synchronous pacing over time using a leadless ventricular pacemaker: Primary results from the AccelAV study. *Heart Rhythm*. 20(1):46–54, 2023. 10.1016/j.hrthm.2022.08.033. 36075532 10.1016/j.hrthm.2022.08.033

[3] Steinwender, C., et al. Atrioventricular synchronous pacing using a leadless ventricular pacemaker: Results from the MARVEL 2 study. *JACC Clin Electrophysiol*. 6(1):94–106, 2020. 10.1016/j.jacep.2019.10.017. 31709982 10.1016/j.jacep.2019.10.017

[4] Arps, K., et al. Optimizing mechanically sensed atrial tracking in patients with atrioventricular-synchronous leadless pacemakers: A single-center experience. *Heart Rhythm O2*. 2(5):455–462, 2021. 10.1016/j.hroo.2021.08.003. 34667960 10.1016/j.hroo.2021.08.003 PMC8505205

[5] Briongos-Figuero, S., et al. Optimizing atrial sensing parameters in leadless pacemakers: Atrioventricular synchrony achievement in the real world. *Heart Rhythm*. 19(12):2011–2018, 2022. 10.1016/j.hrthm.2022.08.007. 35952980 10.1016/j.hrthm.2022.08.007

[6] Neugebauer, F., et al. Leadless atrioventricular synchronous pacing in an outpatient setting: Early lessons learned on factors affecting atrioventricular synchrony. *Heart Rhythm*. 19(5):748–756, 2022. 10.1016/j.hrthm.2021.12.025. 34971817 10.1016/j.hrthm.2021.12.025

[7] Volosin, K. J., D. V. Exner, M. S. Wathen, L. Sherfesee, A. P. Scinicariello, and J. M. Gillberg. Combining shock reduction strategies to enhance ICD therapy: A role for computer modeling. *J. Cardiovasc. Electrophysiol.* 22(3):280–289, 2011. 10.1111/j.1540-8167.2010.01918.x. 20958831 10.1111/j.1540-8167.2010.01918.x

